# Father involvement in early child-rearing and behavioural outcomes in their pre-adolescent children: evidence from the ALSPAC UK birth cohort

**DOI:** 10.1136/bmjopen-2016-012034

**Published:** 2016-11-22

**Authors:** Charles Opondo, Maggie Redshaw, Emily Savage-McGlynn, Maria A Quigley

**Affiliations:** Policy Research Unit in Maternal Health and Care, National Perinatal Epidemiology Unit, Nuffield Department of Population Health, University of Oxford, Oxford, UK

**Keywords:** fathers involvement, child-rearing, behavioural outcomes, ALSPAC

## Abstract

**Objective:**

To explore the nature of paternal involvement in early child-rearing adopting a social developmental perspective, and estimate its effect on behavioural outcomes of children aged 9 and 11 years.

**Setting:**

The data come from the Avon Longitudinal Study of Parents and Children (ALSPAC) cohort recruited in the former county of Avon in the southwest of England.

**Participants:**

Out of the 14 701 children in this cohort who were alive at 1 year, 10 440 children were living with both parents at 8 months and were therefore eligible. Outcome data were available for 6898 children at 9 years and 6328 children at 11 years.

**Main exposure:**

Paternal involvement was measured using factor scores obtained through factor analysis of fathers’ responses on their participation in, understanding of, and feelings about their child's early upbringing.

**Outcome:**

Behavioural problems were measured using the Strengths and Difficulties Questionnaire (SDQ) total difficulties score.

**Results:**

3 factors were identified in the factor analysis: Factor 1 described fathers’ emotional response to the child; factor 2 measured the frequency of fathers’ involvement in domestic and childcare activities; factor 3 characterised fathers’ feelings of security in their role as parent and partner. Children of fathers with high scores on factors 1 and 3 had 14% (OR 0.86, 95% CI 0.79 to 0.94, p=0.001) and 13% (OR 0.87, 95% CI 0.79 to 0.96, p=0.006), respectively, lower adjusted odds of behavioural problems at 9 years. Factors 1 and 3 were associated with comparable reduction in adjusted odds of behavioural problems at 11 years (OR 0.89, 95% CI 0.81 to 0.98, p=0.017 and OR 0.89, 95% CI 0.81 to 0.99, p=0.034, respectively). Factor 2 was not associated with the outcome.

**Conclusions:**

Psychological and emotional aspects of paternal involvement in children's early upbringing, particularly how new fathers see themselves as parents and adjust to the role, rather than the quantity of direct involvement in childcare, is associated with positive behavioural outcomes in children.

**Strengths and limitations of this study**

- The study is based on a large sample derived from detailed cohort data.
- A rigorous approach has been adopted to explore the multifaceted nature of the main exposure.
- The study highlights the role of fathers in child development, which has been relatively under-researched.
- Findings are based on observational data which is often subject to unmeasured confounding.
- The main exposure and outcome are based on self-report, which may be subject to bias.

## Introduction

In most societies, the involvement of fathers in child-rearing has traditionally been framed as the role of ‘provider’,^1^ ^2^ with mothers doing most of the task-oriented caring and nurturing of children. However, in recent decades social changes including the rapid increase in the proportion of working mothers^3–5^ and changes in employment regulations such as increased paternity leave have resulted in a shift towards more of the direct parenting duties being shared by both parents.^6^ Understanding the nature and effect of fathers' involvement on the health and well-being of children could therefore help inform policies aimed at improving family psychological and health outcomes.

The nature of parenting in a child's early years is thought to play an important role in influencing the child's immediate and long-term well-being and mental health, including social development,^7^ ^8^ and cognitive and educational outcomes.^9^ The years of middle childhood preceding adolescence represent a developmental stage that is marked by rapid physical growth, cognitive change and the development of social awareness and skills.^10^ The nature and extent of fathers' involvement in parenting may change over the course of a child's life. However, early paternal involvement is often associated with continuing engagement and may be a proxy measure of overall engagement.^11–13^ Early parenting can also affect outcomes later in life.^14^ ^15^ For these reasons we were interested in whether fathers' involvement early in their child's life was associated with the child's later mental health and social development. We focused on the child's behaviour as a component of mental health because of its strong link with cognitive^16^ and educational outcomes.^17^

Paternal involvement, as with maternal involvement and that of the family more broadly, is multifaceted.^18^ ^19^ It can be characterised by: fathers' *accessibility* to their children measured by their frequency of contact with the child,^20^ ^21^ co-residence with the child^20^ or even presence at the child's birth;^22^ their *engagement* in childcare activities such as playing, feeding and bathing;^23–25^ and their demonstration of *responsibility* in providing for the material^25^ and emotional^26^ needs of their children. Nevertheless, many studies have tended to characterise paternal involvement as a unidimensional construct.^27^ The failure to adopt a more multidimensional approach may explain why the evidence for its effect on mental health outcomes in children is unclear.

In this study we sought to first identify the multidimensional aspects of paternal involvement before investigating their potential influence on pre-adolescent children's behaviour. We then explored the relationship between paternal involvement in the child's upbringing at 8 weeks and 8 months postnatally and child behavioural outcomes at age 9 and 11 years, hypothesising that greater early paternal involvement would be associated with positive behavioural outcomes.

## Methods

### Data

Data were drawn from the Avon Longitudinal Study of Parents and Children (ALSPAC) cohort.^28^ ^29^ It is based on a sample of children born to mothers living in the former county of Avon in the southwest of England between April 1991 and December 1992. A detailed description of the sample profile has been provided elsewhere^30^ ^31^ and the study website contains details of all the data that is available through a fully searchable data dictionary.^32^

**Figure 1:**
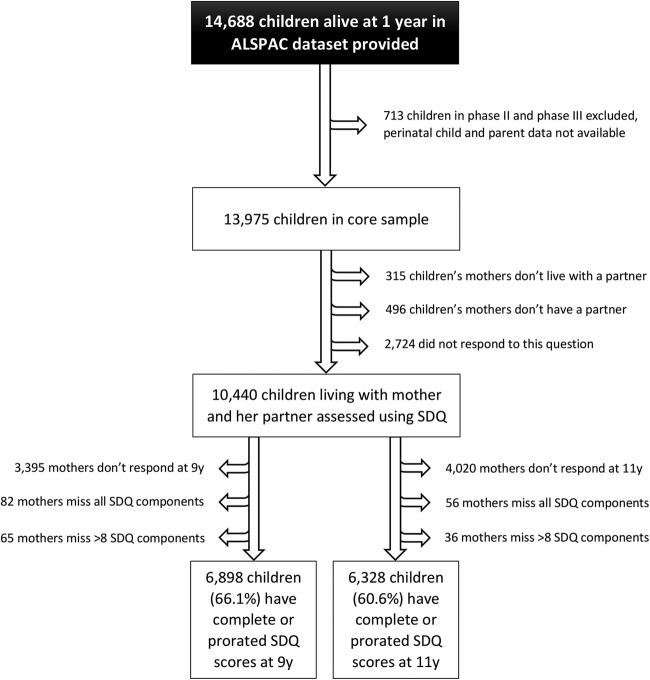
Sample profile of the children included in the analysis. ALSPAC, Avon Longitudinal Study of Parents and Children; SDQ, Strengths and Difficulties Questionnaire.

There were 14 701 children in the total sample who were alive at 1 year. Data on 14 688 term singletons and twins who were alive at age 1 year were provided for these analyses and 13 observations on higher-order multiple births omitted from our data to preserve confidentiality. We also excluded 713 children recruited retrospectively when the children in the original core sample were around 7 years old (phase II and phase III recruitment) and 3535 children whose mothers did not live with (or report living with) a partner at 8 months. Out of the remaining 10 440 eligible children, the analysis was based on 6898 and 6328 children whose mothers completed the Strengths and Difficulties Questionnaire (SDQ) at age 9 and 11 years, respectively (figure 1).

### Outcome, exposure and potential confounder variables

Data were collected using self-completion questionnaires sent to mothers and their partners after recruitment and when the child was aged 8 weeks, 8 months, 9 years and 11 years. The questionnaires asked about mental health, parenting and childcare, behaviour, socioeconomic status (SES) of parents, and child development.

The outcome was the child's behaviour measured by SDQ completed by the mother.^33^ This tool has five subscales, namely ‘emotional symptoms’, ‘conduct problems’, ‘hyperactivity’, ‘peer relationship problems’ and ‘prosocial behaviour’. Each scale is made up of five items giving a total of 25 items. The items are statements about psychological attributes, some positive (eg, ‘considerate of other people's feelings’ and ‘kind to younger children’) and others negative (eg, ‘restless, overactive, cannot stay still for long’ and ‘nervous or clingy in new situations, easily loses confidence’). One of three ordered responses are given to each question: 0 (‘not true’), 1 (‘somewhat true’) or 2 (‘certainly true’). These are then summed up to obtain the scale scores and the total SDQ score. In our analyses we used the SDQ total difficulty score derived by summing up the scores of the first four scales of the SDQ^34^ measured at age 9 and 11 years.

Paternal involvement was measured by asking fathers to rate their level of agreement—on 3–6-point ordinal scales—with items developed exclusively for the study by the ALSPAC study team and not drawn from an existing scale. We identified and selected 58 statements based on paternal report which reflected direct care and associated household tasks, fathers' attitudes to parenting, relationship with child, and fathers' moods and feelings in the post-partum period at 8 weeks (37 items) and 8 months (21 items) after the birth of the child.

Data on other factors measured after recruitment which, according to the literature,^35–40^ were potentially associated with the main outcome and/or exposure were also obtained including parental age, level of education (O-level/Certificate of Secondary Education (CSE)/vocational training, A-level or university degree), parity, depression symptoms measured 8 weeks postnatally on the Edinburgh Postnatal Depression Scale (EPDS),^41^ SES derived from self-reported occupation using the Computer Assisted Structured Coding Tool (CASCOT) and coded into quintiles from 1 (lowest) to 5 (highest),(Warwick Institute for Employment Research, “Computer Assisted Structured Coding Tool (CASCOT).” 2000.), number of hours worked in the current or most recently held job, and child's age and gender.

### Analysis

Exploratory factor analysis (EFA) was performed on the items measuring fathers' direct care and associated household tasks, attitudes, relationship with child, and moods and feelings in the early months. Factors identified in this analysis were deemed to represent key aspects of paternal involvement. Next, confirmatory factor analysis (CFA) was undertaken to check whether the factor structure identified during EFA was a good fit for the data. To improve the fit of the CFA model items that loaded on more than one factor were removed from further analysis, as were items with low factor loadings (<0.3), because they were deemed to be poor measures of the underlying construct.^42^ Once a well-fitting factor structure was identified, factor scores for each of the identified aspects of paternal involvement were calculated. Factor scores are standard normal estimates of each individual's relative position in the continuum of the latent characteristic being measured. They were calculated as the maximum of the posterior distribution (MAP) of the latent factors given the observed and unobserved responses corresponding to the individual.^43^ Factor scores so derived yield unbiased regression slopes when used as predictors in models.^44^ Labels corresponding to the construct represented by the items underpinning each factor were then attributed to the factor scores to facilitate interpretation of the results.

To explore the association between paternal involvement and behavioural outcomes a two-step procedure was undertaken. First, univariable-ordered logistic regression models of SDQ total difficulties scores on the factor scores and paternal, maternal and child covariates were fitted to identify the factors which were associated with the outcome. Next, all factors found to be independently associated with SDQ score (likelihood ratio p values <5%) were included in a multivariable model, retaining only those factors for which there was still evidence of association with the outcome even after adjustment. Potential differences in association for boys and girls were explored by fitting and testing interaction terms for gender. Separate models were fitted for SDQ measured at 9 and 11 years.

Missing data were dealt with in three ways. First, the MAP estimation of factor scores allowed for the partial recovery of information contained in unobserved paternal involvement items. Second, missing data in SDQ component items were handled by prorating scale scores for up to two missing responses out of the five items in each scale.^34^ It is a single imputation procedure which replaces an individual's missing item scores with the mean score for the scale to which the missing item belongs. Third, missing values of covariates in the multivariable regression modelling were assumed to be missing completely at random. Multiple imputation was performed to recover the information contained in these missing values, with 10 imputed datasets used to obtain the final estimates.

The proportional odds assumption underpinning the ordered logistic regression models was examined using the Brant test with χ^2^ p values <5% interpreted as a violation of this assumption.^45^ Data management and manipulation were performed using Stata V.13 (StataCorp. “Stata 13.” StataCorp LP, College Station, TX: 2013.). Analyses were run in Stata V.13 and MPlus V.7 (Muthén LK, Muthén BO. MPlus 7. Los Angeles, CA: Muthén & Muthén, 2015).

## Results

The characteristics of the samples at 9 and 11 years were very similar (table 1). Overall there were slightly more boys than girls. Fathers were on average aged 31 years and educated to O-level or CSE (examinations completed in the final year of compulsory schooling at age 16 years) or had some vocational (skills) training. They reported working about 45 hours/week in their current or most recent job around the time when the child was 8 months old and had low depression scores on the EPDS. Mothers were on average 2 years younger than the fathers, were educated to a similar level, had slightly higher EPDS scores than fathers, and had one child prior to the one included in the study. Parents tended to be in similar SES categories. Most children had low total difficulties scores, with medians of 6 and 5 at the two respective time points.

**Table 1:** Characteristics of the parents and children included in the samples at the two time points

	9 years (n=6898)	11 years (n=6328)
*Fathers*		
Age in years 18 weeks after birth of child, mean (SD)	31.27 (5.4)	31.30 (5.4)
Highest level of education, n (%)		
O-level, CSE* or vocational	3021 (43.8%)	2758 (43.6%)
A-level†	1482 (21.5%)	1366 (21.6%)
University degree	1048 (15.2%)	1002 (15.8%)
Missing	1347 (19.5%)	1202 (19.0%)
Hours worked per week, mean (SD)	44.83 (9.8)	44.77 (9.9)
EPDS score, median (IQR)	3 (1–6)	3 (1–6)
SES‡ category, n (%)		
1—lowest	138 (2.0%)	122 (1.9%)
2	499 (7.2%)	453 (7.2%)
3	2516 (36.5%)	2297 (36.3%)
4	2361 (34.2%)	2184 (34.5%)
5—highest	875 (12.7%)	823 (13.0%)
Missing	509 (7.4%)	449 (7.1%)
*Mothers*		
Age in years at birth of child, mean (SD)	29.30 (4.4)	29.33 (4.4)
Highest level of education, n (%)		
O-level, CSE or vocational	2564 (37.2%)	2317 (36.6%)
A-level	1665 (24.1%)	1564 (24.7%)
University degree	1413 (20.5%)	1329 (21.0%)
Missing	1256 (18.2%)	1118 (17.7%)
Parity, median (IQR)	1 (0–1)	1 (0–1)
EPDS score, median (IQR)	5 (2–8)	5 (2–8)
SES category, n (%)		
1—lowest	86 (1.3%)	79 (1.3%)
2	443 (6.4%)	417 (6.6%)
3	2844 (41.2%)	2613 (41.3%)
4	2099 (30.4%)	1926 (30.4%)
5—highest	441 (6.4%)	423 (6.7%)
Missing	985 (14.3%)	870 (13.6%)
*Children*		
Mean age difference in months, mean (SD)	0.00 (5.8)	0.00 (5.8)
Gender, n (%)		
Boys	3499 (50.7%)	3170 (50.1%)
Girls	3399 (49.3%)	3158 (49.9%)
SDQ total difficulties score, median (IQR)	6 (3–9)	5 (3–9)

*O-level and CSE were the national exams which students in England sat in their last year of compulsory school education at age 16.

†A-levels are preuniversity examinations.

‡SES is derived from the CASCOT.

CASCOT, Computer Assisted Structured Coding Tool; EPDS, Edinburgh Postnatal Depression Scale; SDQ, Strengths and Difficulties Questionnaire; SES, socioeconomic status.

There were complete responses to the 58 paternal involvement questions for between 4560 and 5381 children at 8 weeks and 8 months. A total of 45 of the 58 items reflecting paternal involvement were included in the EFA. Reasons for exclusion of 13 items were: high uniqueness coefficients of >0.9 affecting 8 items; low loadings on the retained factors affecting 1 item; large amounts of missing data—more than 50%—affecting 3 items; and similarity between 2 items leading to one of them being dropped. Table 2 presents the results of the EFA.

**Table 2:** Exploratory factor analysis on the indicators of paternal involvement

	Rotated loadings*		
Indicator	Factor 1	Factor 2	Factor 3
Helped with shopping since birth		0.4891	
Helped with cleaning home since birth		0.7112	
Helped with meal preparation since birth		0.7013	
Helped with washing up since birth		0.6077	
Helped with housework since birth		0.7573	
Helped with cooking meals since birth		0.6882	
Helped with clothes wash since birth		0.5878	
How frequently partner changes nappy per week?		0.5178	
How frequently partner bathes child per week?		0.4293	
How frequently partner plays with child per week?	0.4324	0.4107	
How frequently partner walks child outside per week?		0.4284	
How frequently partner puts child to bed per week?		0.4225	
How frequently partner feeds/helps at night per week?		0.4294	
Mum excludes partner from childcare			0.7346
Feel confident with child	0.3857		−0.3201
Feel mum does not trust partner with child			0.6817
Happy with the way mum brings up child	0.3652		−0.4507
Happy with the way partner brings up child	0.5094		−0.4468
Making a strong bond with child	0.6147		
My stress is a bad influence on child	−0.3728		0.4486
Home is woman's place, no part for me		−0.3643	0.3871
Partner always getting under mum's feet			0.4928
Mum dislikes partner being involved with child			0.7315
Partner guilty for not enjoying child	−0.5480		0.4373
Partner regrets having child	−0.6553		
Partner regrets lack of experience of children			0.3160
This child has made partner more fulfilled	0.6465		
Parenthood has made partner and mum closer	0.5038		
Mum no longer gives partner attention			0.5413
Feel hurt by attention mum gives child			0.5760
Partner well prepared for birth and childcare			−0.3166
Partner enjoys getting home to see mum and child	0.6307		
Enjoy the baby	0.8294		
Preferred not to have had baby	−0.5550		
Feel confident with baby	0.5776		
Dislike mess surrounding baby	−0.3707		
Pleasure watching baby develop	0.8240		
Find baby crying unbearable	−0.3836		
Constantly unsure whether doing right thing			0.3160
Feel should enjoy baby but am not	−0.6869		
No time to self	−0.4583		
Baby made feel more fulfilled	0.7315		
Feel babies are fun	0.8185		
Talking to baby is important	0.4211		
Cuddling baby is very important	0.4612		

*Displaying only loadings >0.3.

Three factors of paternal involvement, explaining 66.0% of the total variance in included items, were identified: items in the first factor, explaining 31.4% of total variance, described fathers' emotional response to the baby and their parenting role; items in the second factor, explaining 17.7% of total variance, measured fathers' level of engagement in domestic and childcare activities; and items in the third factor, explaining 16.9% of total variance, characterised fathers' security in their role as a parent and partner. CFA, which excluded cross-loading items (since this implies that they poorly discriminate between factors^46^), showed this factor structure to be an acceptable representation of the correlations between items in the data according to the model's comparative fit index (CFI), Tucker-Lewis index (TLI) and root mean square error of approximation (RMSEA) (CFI=0.916, TLI=0.911, RMSEA=0.065).

Unadjusted ordered logistic regression showed strong evidence that the first and third factor scores were associated with the outcome (table 3). Children of fathers whose responses corresponded to higher factor 1 scores had 21% and 19% reductions in proportional odds of higher SDQ total difficulty scores at ages 9 and 11, respectively, and children of fathers whose responses corresponded to higher factor 3 scores had 28% reduction in proportional odds of higher SDQ total difficulty scores at both times. Other factors were also found to be associated with the outcome. Specifically, higher parental age, level of education and SES category were associated with reduced proportional odds of higher behavioural difficulty scores, while more hours worked per week, higher EPDS scores, child's age and male gender were associated with poorer behavioural outcomes.

**Table 3:** Unadjusted and adjusted proportional ORs for the effect of paternal involvement on SDQ scores at ages 9 and 11 years, with 95% CIs and p values

	9 years		11 years	
Paternal involvement factor scores	Unadjusted [n=5717]	Adjusted* [n=6223]	Unadjusted [n=5262]	Adjusted* [n=5500]
Factor 1: “emotional response to baby and parenting”	OR 0.79 95% CI 0.73 to 0.86 p value <0.001	OR 0.86 95% CI 0.79 to 0.94 p value 0.001	OR 0.81 95% CI 0.74 – 0.88 p value <0.001	0.89 95% CI 0.81 – 0.98 p value 0.017
Factor 2: “engagement in domestic and childcare activities”	OR 1.01 95% CI 0.90 – 1.14 p value 0.854	–	OR 1.09 95% CI 0.97 – 1.24 p value 0.160	–
Factor 3: “security in role as parent and partner”	OR 0.72 95% CI 0.66 – 0.78 p value <0.001	OR 0.87 95% CI 0.79 – 0.96 p value 0.006	OR 0.72 95% CI 0.66 – 0.79 p value 0.001	OR 0.89 95% CI 0.81 – 0.99 p value 0.034

*Adjusting for paternal and maternal EPDS score, parity, maternal age, family SES score, child's age and child's gender

EPDS, Edinburgh Postnatal Depression Scale; SDQ, Strengths and Difficulties Questionnaire; SES, socioeconomic status.

Multivariable models adjusting for these potential confounders were fitted, with multiple imputation of missing values of covariates. The adjusted proportional odds of higher behavioural problems scores were 14% lower at 9 years and 11% lower at 11 years per unit increase in factor 1 scores and 13% lower at 9 years and 11% lower at 11 years per unit increase in factor 3 scores, comparing children of the same age and gender, family size, SES, who were exposed to the same level of parental depression (table 3). There was no evidence of a difference in the effect of paternal involvement in boys versus girls; p values for interaction of child's gender with factor 1 and with factor 3 were 0.907 and 0.864, respectively, in models of the outcome at 9 years, and 0.189 and 0.918, respectively, at 11 years. The proportional odds assumption was not violated in these models; Brant test p values were 0.078 and 0.050 for models on data at 9 years and 0.316 and 0.514 on models at 11 years.

## Discussion

This analysis of data from over 6000 fathers and children in the south-west of England characterised the nature of paternal involvement in early child upbringing and explored its effects on behavioural outcomes in pre-adolescent children. We found that the children of fathers whom we characterised as having a positive emotional response to parenting and a sense of security in their role as a parent and partner early in the child's life—corresponding to higher scores on factor 1 and factor 3, respectively—were less likely to exhibit behavioural problems at 9 and 11 years of age. These factors may reflect ways of behaving and interacting that are a marker of favourable parental characteristics and positive parenting in the longer term. Our analyses also show that the amount of paternal involvement with childcare and household tasks such as shopping, cleaning, cooking, and childcare activities was not associated with later child behavioural problems. This may be because provision of more direct childcare by fathers may simply reflect temporary circumstances and needs, for example, the absence of extended family support and type of partner employment.

Our findings are consistent with previous suggestions that paternal involvement may encompass different aspects of how fathers interact with their children and partners, and these aspects potentially differ in terms of how they manifest themselves in father–child interactions, and also in their effects on child outcomes. While paternal involvement is broadly associated with a variety of positive outcomes for children—either directly or indirectly such as through resource-related benefits on health, education and general well-being—previous evidence for whether it contributes to mental health outcomes has not been consistent. For example, paternal presence and involvement in childcare,^47^ and shared activities, supportive behaviour and feelings of affection towards children^48^ have been shown to be associated with lower likelihood of behavioural problems in children aged 3 and 5–18 years, respectively. Similarly, fathers' involvement in childcare has been linked to cognitive outcomes in 2–6-year-olds.^49^ ^50^ However, as in this study, others have found little or no evidence of an effect of fathers' involvement in childcare on behavioural outcomes,^49–51^ suggesting that the amount of paternal involvement in these activities may not be as important for this outcome as the type of involvement and attitudes towards parenting.^26^

Although we measured paternal involvement as a multidimensional construct, our approach did not mirror the three defining elements of an involved father—engagement, accessibility and responsibility—originally proposed by Lamb *et al* in 1985.^19^ We included in our analysis only children whose fathers (or father-figures) were present in the early years but who varied in terms of their level of financial contribution to their families, and as such were not able to distinguish between different levels of fathers' financial contributions. However our analysis adjusted for socioeconomic differences between families based on the likely income level of both parents. This may be a better way of dealing with financial contribution in general since it is increasingly common for either or both parents to be breadwinners, and as such fathers making little or no financial contribution to their family would not necessarily imply a of lack of responsibility. Further deviating from the original Lamb-Pleck conceptual framework, our analysis suggests that paternal involvement may extend beyond activities encompassing direct childcare to associated activities and fathers' attitudes towards the child and themselves as parents.

Positive parenting by fathers may contribute to good outcomes in children in a number of ways. Involved fathers may influence children indirectly by being a source of instrumental and emotional support to mothers who provide more of the direct care for children.^26^ The potential positive effect of this on mothers' well-being and parenting strategies^52^ may then lead to better outcomes in children.^53^ There is evidence that fathers' involvement can also alleviate the impact of factors such as maternal depression which are known to increase children's risk of behavioural problems.^54^ Greater paternal involvement may also lead to or be a manifestation of a happy and cohesive family,^55^ and this may bring about better outcomes in children. Social and cultural differences within and across societies may limit the generalisability of these findings.

There are several limitations to this study. The nature of paternal involvement continues to evolve over time, and because this study is based on a cohort which was born 25 years ago there may be a limit to how generalisable its findings are to the present day. This was an analysis of observational data, and although we have adjusted for several factors known to be associated with child behaviour, we cannot rule out the possibility of residual confounding. There were a number of sources of potential bias. For example, child behavioural outcome measures reported by mothers could have been influenced by the mothers' own mental health or their attitudes to their children which in turn could be associated with paternal involvement.^56–58^ However in these data parent-level predictors preceded child-level outcomes by up to a decade, which is likely to have weakened this bias. We used fathers' own reports of their involvement, and this may have further reduced the potential for information bias that may arise with mother-reported measures of paternal involvement. The large sample size, detailed data on fathers' involvement and our rigorous approach to the analysis were also a strength, particularly in exploring the multifaceted nature of paternal involvement. Finally, a number of plausible mechanisms of the association between paternal involvement and child behaviour support the observed associations.

While acknowledging the impact of both parents on children's development and outcomes, there is scope for further research to address some related questions concerning the role of fathers. These include exploring the effect of paternal involvement on other mental health outcomes such as identity, self-esteem, emotional and social development, and how they vary over time. For example, the effect of paternal involvement on child behaviour may become less important over time especially in the adolescent period when peer relationships and other factors such as age and gender start being more influential. This could be explored using growth curve models, but would require further outcome data points.

## Conclusion

The findings of this research study suggest that it is psychological and emotional aspects of paternal involvement in a child's infancy that are most powerful in influencing later child behaviour and not the amount of time that fathers are engaged in childcare or domestic tasks in the household. How new fathers see themselves as parents, how they value their role as a parent and how they adjust to this new role, rather than the amount of direct involvement in childcare in this period, appears to be associated with positive behavioural outcomes in children.
